# Lean Metabolic-Dysfunction-Associated Steatotic Liver Disease (MASLD): Pathophysiology, Diagnostic Challenges, Clinical Outcomes, and Management

**DOI:** 10.3390/diseases14050173

**Published:** 2026-05-15

**Authors:** Noor Albusta, Sara Isa, Hussain Alrahma

**Affiliations:** 1 Department of Internal Medicine, Lahey Hospital and Medical Center, Burlington, MA 01805, USA; 2 School of Medicine, Royal College of Surgeons in Ireland—Medical University of Bahrain, Adliya P.O. Box 15503, Bahrain; 19201227@rcsi.com; 3 Department of Gastroenterology and Hepatology, Salmaniya Medical Complex, Manama P.O. Box 12, Bahrain; hrahma1@hospitals.gov.bh

**Keywords:** lean MASLD, metabolic-dysfunction-associated steatotic liver disease, visceral adiposity, insulin resistance, liver fibrosis, cardiometabolic risk, sarcopenia

## Abstract

Background/Objectives: Lean metabolic dysfunction-associated steatotic liver disease (lean MASLD) is an increasingly recognized phenotype occurring in individuals with normal body mass index (BMI), despite clinically important hepatic and cardiometabolic risk. This narrative review summarizes current evidence on its epidemiology, pathophysiology, diagnostic challenges, clinical outcomes, and management. Methods: A narrative literature review was conducted using PubMed, Embase, and Cochrane Library from database inception to March 2026. Relevant studies on lean MASLD/lean NAFLD, including cohort studies, meta-analyses, clinical trials, consensus statements, and practice guidelines, were prioritized. Results: Lean MASLD reflects interactions between visceral adiposity, insulin resistance, genetic susceptibility, sarcopenia, dietary and lifestyle factors, vitamin D deficiency, and gut microbiome alterations. Diagnosis is challenging because BMI and aminotransferase levels may underestimate metabolic vulnerability, MASH, or clinically significant fibrosis. Available data suggest increased liver-related events, liver-related mortality, and all-cause mortality compared with individuals without steatotic liver disease, although comparisons with non-lean MASLD remain heterogeneous. Resmetirom and semaglutide have expanded treatment options for noncirrhotic MASH with moderate to advanced fibrosis, but lean patients are underrepresented in pivotal trials. Conclusions: Lean MASLD is an underrecognized but clinically important phenotype. Earlier recognition, fibrosis risk stratification, sarcopenia assessment, cardiometabolic optimization, and lean-specific therapeutic research are needed to improve outcomes.

## 1. Introduction

Metabolic-dysfunction-associated steatotic liver disease (MASLD) is the most prevalent chronic liver condition worldwide, affecting more than one-third of adults. It is also a leading cause of liver-related morbidity and mortality [1]. Historically termed nonalcoholic fatty liver disease (NAFLD), the condition was renamed in 2023 following a Delphi consensus process to better reflect its metabolic underpinnings and reduce stigma [2]. MASLD encompasses a spectrum ranging from simple hepatic steatosis to metabolic-dysfunction-associated steatohepatitis (MASH), progressive fibrosis, cirrhosis, and hepatocellular carcinoma (HCC) [2,3].

Conventional understandings of MASLD have emphasized its association with obesity and metabolic syndrome. However, accumulating evidence demonstrates that a substantial proportion of individuals with MASLD have a normal BMI, a phenotype termed “lean NAFLD,” “non-obese NAFLD,” and “lean MASLD” [4,5]. This observation challenges the assumption that hepatic steatosis is exclusively a disease of overweight and obesity and has prompted investigation into the distinct pathophysiology, history, and clinical implications of lean MASLD.

The clinical relevance of lean MASLD extends beyond its prevalence. Studies suggest that affected individuals may harbor significant metabolic dysfunction despite normal weight, including visceral adiposity, insulin resistance, dyslipidemia, and sarcopenia [6,7]. Moreover, cohort and meta-analytic data indicate that lean MASLD is not benign: liver-related events, liver-related mortality, and all-cause mortality may be increased compared with lean metabolically healthy individuals and, in some cohorts, non-lean MASLD populations [8,9,10]. These findings have important implications for screening, diagnosis, and management, as reliance on BMI alone may delay recognition and intervention.

Despite growing awareness, lean MASLD remains underdiagnosed and understudied. Contemporary U.S. data suggest that a substantial proportion of lean MASLD cases remain undiagnosed, emphasizing the limitations of weight-centered care [11]. Diagnostic algorithms developed for broader MASLD populations may not adequately capture the unique features of lean disease, and therapeutic evidence specific to this phenotype remains limited. Furthermore, the biological heterogeneity underlying lean MASLD—encompassing genetic predisposition, body composition abnormalities, dietary patterns, sleep and lifestyle factors, and gut microbiome alterations—suggests that a one-size-fits-all approach to management is unlikely to be optimal [12,13].

The therapeutic landscape for MASH has recently changed. Resmetirom, a liver-directed thyroid hormone receptor-β agonist, received accelerated U.S. Food and Drug Administration (FDA) approval in March 2024 for adults with noncirrhotic nonalcoholic steatohepatitis/MASH with moderate to advanced fibrosis. Semaglutide received accelerated FDA approval in August 2025 for adults with noncirrhotic MASH with moderate to advanced fibrosis [14,15,16,17]. These treatments are the first pharmacological therapies specifically indicated for MASH, altering the management paradigm. However, their applicability to lean MASLD remains uncertain, as lean patients were underrepresented in trials.

In this narrative review, we synthesize current knowledge on lean MASLD, addressing its epidemiology, pathophysiological mechanisms, diagnostic challenges, clinical outcomes, and treatment in light of recent advances. By highlighting this phenotype’s distinct features, we seek to inform clinical practice and identify priorities for future research.

This study was designed as a narrative review rather than a systematic review. A structured literature search was performed using PubMed, Embase, and Cochrane Library from database inception to March 2026. Search terms included combinations of “lean MASLD,” “lean NAFLD,” “non-obese NAFLD,” “metabolic dysfunction-associated steatotic liver disease,” “metabolic dysfunction-associated steatohepatitis,” “MASH,” “NASH,” “visceral adiposity,” “sarcopenia,” “insulin resistance,” “PNPLA3,” “TM6SF2,” “gut microbiome,” “non-invasive tests,” “FIB-4,” “VCTE,” “ELF,” “MRI-PDFF,” “resmetirom,” and “semaglutide.” Studies using prior “NAFLD/NASH” terminology were included when the population and disease phenotype were relevant to current MASLD/MASH concepts [2,3,4].

Original cohort studies, population-based analyses, meta-analyses, clinical trials, consensus statements, and society guidelines were prioritized. Studies were included if they addressed lean or non-obese steatotic liver disease epidemiology, mechanisms, diagnostic evaluation, clinical outcomes, or management. Studies were excluded if they only focused on pediatric populations, alcohol-related liver disease, viral hepatitis, or secondary causes of steatosis without direct relevance to lean MASLD. Because this was a narrative review, formal risk of bias scoring was not performed; however, conflicting findings were appraised qualitatively with attention to study design, population characteristics, diagnostic definitions, adjustment strategy, and follow-up duration.

Because many foundational studies were published before the nomenclature transition, the terms “NAFLD” and “NASH” are retained only when referring to historical study populations, trial names, regulatory language, or original article terminology; otherwise, MASLD and MASH are used throughout.

## 2. Epidemiology of Lean MASLD

### 2.1. Global Prevalence and Regional Variation

While the epidemiology of lean MASLD has been increasingly characterized over the last two decades, estimates vary depending on the population studied, the method used to identify hepatic steatosis, the definition of metabolic dysfunction, alcohol exclusion thresholds, and BMI cutoffs used to define “lean” status. Earlier studies used NAFLD criteria requiring hepatic steatosis after exclusion of significant alcohol intake and other secondary causes, whereas contemporary MASLD criteria require hepatic steatosis and at least one cardiometabolic risk factor [2,3,4]. Diagnostic methods have also differed across studies, including ultrasound, controlled attenuation parameter (CAP), magnetic-resonance-imaging-derived proton density fat fraction (MRI-PDFF), histology, International Classification of Diseases codes, and composite non-invasive definitions. Most studies define lean MASLD as hepatic steatosis in individuals with a BMI <25 kg/m^2^ in Western populations or a BMI <23 kg/m^2^ in Asian populations, consistent with ethnicity-specific definitions of normal weight [4,18,19]. These methodological differences partly explain the variability in reported prevalence and outcomes across cohorts.

Meta-analyses estimate that lean individuals account for approximately 10–20% of all MASLD/NAFLD cases globally, with pooled prevalence estimates ranging from approximately 5% to 12% depending on definitions and diagnostic methods [5]. Prevalence differs substantially by geographic region. Asian populations, particularly those from East and South Asia, have higher rates than Western cohorts, attributed to differences in body fat distribution, genetic susceptibility, dietary patterns, and lower BMI thresholds for metabolic risk [18,19,20]. In contrast, a stronger association between MASLD and obesity has been found in Western populations, although lean disease remains clinically significant [5,6].

Population-based studies in the United States have provided important contemporary data. An NHANES 2017–2020 analysis estimated a meaningful national burden of lean MASLD among U.S. adults with normal BMI and found that a substantial proportion of affected individuals were undiagnosed [11]. MASLD prevalence among lean individuals increased with worsening glycemic status, indicating the importance of cardiometabolic evaluation even when BMI is normal [11]. Data from China, India, and Japan also suggest that lean or non-obese MASLD contributes substantially to the global burden of liver disease [20,21,22].

### 2.2. Demographic and Clinical Characteristics

The demographic profile of lean MASLD differs in several respects from that of non-lean disease. Some studies suggest a higher proportion of females among lean MASLD patients compared with obese MASLD, although this is inconsistent across cohorts [5,23]. While age distribution appears broadly similar, younger Asian cohorts may be disproportionately affected [24]. Lean individuals with MASLD are more commonly of Hispanic or Asian origin in some cohorts, likely driven in part by genetic susceptibility and differences in body fat distribution [23,25].

Clinically, individuals with lean MASLD often lack the classic phenotypic markers that prompt suspicion of metabolic liver disease. Waist circumference may be normal or only mildly elevated, and overt features of metabolic syndrome may be absent [26]. Nevertheless, careful evaluation frequently reveals subclinical metabolic abnormalities, including insulin resistance, elevated fasting glucose, atherogenic dyslipidemia, and increased visceral adipose tissue on imaging [6,7,27]. These observations highlight the limitations of BMI as a sole indicator of metabolic health and the need for more comprehensive risk assessment in lean individuals.

### 2.3. Temporal Trends and Projected Burden

The incidence of lean MASLD appears to be increasing in parallel with rising rates of MASLD overall. It is unclear whether this reflects true epidemiological change driven by shifting dietary patterns, sedentary behavior, sleep disruption, and environmental exposures or improved detection due to greater clinical awareness. Modeling studies project a rising burden of MASLD and advanced fibrosis in the coming decades, with important implications for liver-related morbidity, transplantation demand, and healthcare utilization [28,29]. Longitudinal cohort studies with standardized case definitions are needed to clarify temporal trends and identify modifiable risk factors for primary prevention.

## 3. Pathophysiology of Lean MASLD

### 3.1. Metabolic Dysfunction Beyond Obesity

The pathogenesis of lean MASLD involves a complex interplay of metabolic, genetic, environmental, and microbiome-related factors that collectively promote hepatic lipid accumulation and inflammation in the absence of generalized adiposity. Metabolic dysfunction is not synonymous with obesity: lean individuals may harbor significant metabolic abnormalities that drive liver disease through mechanisms that are distinct from and yet overlap with those operative in obese MASLD [30,31].

### 3.2. Visceral Adiposity and Body Composition

A key insight from body composition studies is that lean MASLD patients often exhibit increased visceral adipose tissue relative to total body fat, a pattern sometimes termed “metabolically obese, normal weight” [25,32]. Visceral adiposity is strongly associated with insulin resistance, systemic inflammation, and atherogenic dyslipidemia, which contribute to hepatic steatosis and disease progression [33]. Imaging studies using computed tomography and magnetic resonance imaging have demonstrated that visceral adipose tissue area may be a stronger predictor of hepatic steatosis than BMI or total body fat mass in lean individuals [34].

In addition to visceral fat accumulation, sarcopenia is an independent risk factor for MASLD and fibrosis progression [35,36]. Muscle tissue plays a critical role in glucose disposal and energy metabolism, and reduced muscle mass may exacerbate insulin resistance and shift substrate flux toward hepatic lipogenesis. Studies have demonstrated that sarcopenia is associated with nonalcoholic fatty liver disease independent of obesity and insulin resistance and is linked to more severe steatohepatitis and fibrosis [36,37]. The combination of increased visceral adiposity and reduced muscle mass may produce a particularly high-risk phenotype in normal-weight individuals.

### 3.3. Insulin Resistance and Hepatic Lipid Metabolism

Insulin resistance is a central driver of MASLD pathogenesis across BMI. In lean individuals, insulin resistance may be tissue-selective and involve adipose tissue, skeletal muscle, and hepatic pathways to differing degrees [30,31]. Insulin-resistant adipose tissue promotes lipolysis, increasing circulating free fatty acids and hepatic lipid uptake. Hyperinsulinemia and carbohydrate excess also activate lipogenic transcription factors, including sterol regulatory element-binding protein-1c and carbohydrate-responsive element-binding protein, increasing de novo lipogenesis [38].

Dietary factors, particularly high intake of fructose and refined carbohydrates, further stimulate hepatic de novo lipogenesis and may be especially relevant in lean populations with diets rich in processed foods despite normal total body weight [38,39]. Mitochondrial dysfunction, oxidative stress, endoplasmic reticulum stress, and inflammatory signaling contribute to hepatocellular injury, ballooning, and fibrogenesis.

### 3.4. Genetic Susceptibility

In this paper, “significant fibrosis” refers to fibrosis stage F2 or higher, “advanced fibrosis” refers to F3 or higher, and “cirrhosis” refers to F4, consistent with conventional histological staging systems used in MASLD/MASH studies [3,4,39]. When cited studies use non-invasive definitions, fibrosis categories are interpreted according to thresholds reported by the original investigators.

Genetic variation plays a prominent role in lean MASLD. Several genetic polymorphisms have been associated with MASLD risk and severity, including variants in PNPLA3, TM6SF2, MBOAT7, GCKR, and HSD17B13 [40,41,42,43]. The PNPLA3 rs738409 I148M variant is the most extensively studied genetic risk factor for steatotic liver disease. Risk allele carriers demonstrate increased hepatic triglyceride content, greater susceptibility to steatohepatitis and fibrosis, and elevated HCC risk [40,44]. The effect of PNPLA3 can be observed in lean individuals, suggesting a role in non-obese disease pathogenesis.

Emerging evidence indicates that genetic architecture may differ between lean and non-lean MASLD, with lean patients potentially enriched for high-risk variants [13,25]. The TM6SF2 E167K variant, which confers susceptibility to MASH and fibrosis but may be associated with lower circulating lipoprotein levels, is particularly relevant because it may partly explain the gap between liver-related and cardiovascular outcomes in select lean MASLD populations [41,45]. Routine genetic testing is not currently recommended in lean MASLD as it does not yet alter standard management, although it may become more relevant as risk-stratified approaches evolve [3,4,19].

Uncommon genetic or metabolic conditions should also be considered in select lean patients, including lipodystrophy, lysosomal acid lipase deficiency, familial hypobetalipoproteinemia, and abetalipoproteinemia [3,4,19].

### 3.5. Gut Microbiome and Metabolic Endotoxemia

Gut microbiome alterations may contribute to lean MASLD through several interrelated pathways, including impaired intestinal barrier integrity, portal translocation of microbial products, altered bile acid metabolism, changes in short-chain fatty acid production, and immune activation within the gut–liver axis [46,47,48]. Increased intestinal permeability may facilitate the translocation of lipopolysaccharides and other pathogen-associated molecular patterns to portal circulation, activating Toll-like receptor signaling, Kupffer cells, hepatic stellate cells, and inflammatory pathways that promote steatohepatitis and fibrogenesis [46,47].

The microbiome in lean MASLD may differ between metabolically healthy lean individuals and patients with obesity-associated MASLD. Altered microbial diversity and shifts in taxa involved in intestinal barrier function, bile acid metabolism, endogenous ethanol production, and inflammatory signaling have been reported, although findings are inconsistent across cohorts [46,47]. Differences in geography, diet, sequencing methods, medication exposure, alcohol thresholds, and MASLD definitions contribute to heterogeneity across studies.

The gut microbiome may be particularly relevant in lean MASLD because affected individuals often lack marked generalized adiposity, suggesting that non-BMI-related drivers, such as diet quality, bile acid signaling, intestinal permeability, and host genetics, may play a larger role. However, causality has not been established. Current evidence does not support routine microbiome testing in clinical practice, and probiotics, prebiotics, synbiotics, fecal microbiota transplantation, and bile-acid-modulating therapies remain investigational [47].

### 3.6. Dietary, Lifestyle, Sleep, and Nutritional Factors

Dietary composition independent of total caloric intake may contribute to lean MASLD risk. High consumption of fructose, sugar-sweetened beverages, refined carbohydrates, saturated fats, and ultra-processed foods can promote de novo lipogenesis, insulin resistance, oxidative stress, and hepatic lipid accumulation [38,39,48]. Conversely, Mediterranean-style dietary patterns rich in vegetables, fruits, legumes, whole grains, fish, olive oil, fiber, and unsaturated fats are associated with improved hepatic steatosis and cardiometabolic risk profiles [49].

Physical inactivity and sedentary behavior further exacerbate metabolic risk even in individuals with normal BMI [50]. Lean individuals with low muscle mass may have reduced capacity for glucose disposal, making resistance exercise particularly important. Exercise may reduce hepatic fat and improve insulin sensitivity even in the absence of major weight loss [51].

Sleep deprivation, obstructive sleep apnea, circadian disruption, and shift work patterns have also been associated with insulin resistance, systemic inflammation, and MASLD risk [52]. Vitamin D deficiency has been linked to hepatic steatosis, metabolic dysfunction, and fibrosis in observational studies, although causality remains uncertain and supplementation data are inconsistent [53]. Other potential factors include low protein intake, sarcopenia, smoking, environmental exposures, and endocrine conditions such as hypothyroidism and polycystic ovary syndrome [3,4,54]. These contributors reinforce the need for individualized assessment rather than weight-centered evaluation alone.

Some lean MASLD patients report relatively healthy dietary habits and active lifestyles, suggesting that genetic and other non-modifiable factors may predominate in select cases [13,25]. This heterogeneity underscores the need for individualized risk assessment and management. Key pathophysiological mechanisms in lean MASLD is summarized in Table 1.

## 4. Diagnostic Challenges in Lean MASLD

### 4.1. Clinical Suspicion and Ascertainment Bias

A fundamental challenge in lean MASLD diagnosis is the low index of clinical suspicion in normal-weight individuals. MASLD screening and case-finding strategies have traditionally targeted populations in which disease prevalence is highest, including patients with obesity, type 2 diabetes, and metabolic syndrome [3,4]. Consequently, lean patients may escape detection until advanced disease stages when symptoms of portal hypertension, hepatic decompensation, or HCC prompt evaluation [55].

This ascertainment bias has important prognostic implications. Studies have reported that lean MASLD patients may present with advanced fibrosis or cirrhosis at diagnosis compared with their obese counterparts, potentially reflecting delayed recognition rather than more aggressive disease biology [9,56]. Heightened awareness among clinicians and incorporation of lean MASLD into risk stratification algorithms are essential to address this problem.

### 4.2. Limitations of BMI as a Diagnostic Criterion

BMI is a crude measure of adiposity that does not distinguish between fat and lean mass or capture regional fat distribution [57]. Individuals with a normal BMI may have a metabolically unfavorable body composition, including elevated visceral adiposity, reduced muscle mass, and ectopic fat deposition in the liver and other organs [25,32,33,34]. These limitations are particularly pronounced in certain ethnic groups—notably South Asians—who develop metabolic complications at lower BMI thresholds [18].

Alternative anthropometric measures, such as waist circumference, waist-to-hip ratio, and waist-to-height ratio, provide better estimates of central adiposity and may improve identification of metabolically at-risk lean individuals [58]. However, these measures are not universally incorporated into routine clinical practice, and standardized cutoffs for lean MASLD screening have not been fully established.

#### Lean MASLD with Normal Aminotransferases

Normal aminotransferase levels do not exclude lean MASLD, MASH, or clinically significant fibrosis. Alanine aminotransferase and aspartate aminotransferase may remain within laboratory reference ranges despite histological steatohepatitis or advanced fibrosis, and reliance on abnormal liver enzymes alone may delay diagnosis [3,19,55]. This is particularly relevant in lean individuals, as clinicians may already have a lower index of suspicion. Therefore, patients with imaging-confirmed steatosis, cardiometabolic risk factors, increased waist circumference, type 2 diabetes, prediabetes, dyslipidemia, hypertension, sarcopenia, or a family history of advanced liver disease should undergo fibrosis risk assessment even when aminotransferases are normal [3,4,19].

### 4.3. Non-Invasive Assessment of Steatosis

MASLD diagnosis requires documentation of hepatic steatosis, exclusion of secondary causes, and—under current nomenclature—the presence of at least one cardiometabolic risk factor [2]. In lean individuals, this may include evidence of insulin resistance, prediabetes, hypertension, dyslipidemia, increased waist circumference, or other cardiometabolic abnormalities, even if overt metabolic syndrome is absent.

Hepatic steatosis is commonly detected through abdominal ultrasound, which is widely available and inexpensive but limited by operator dependence and reduced sensitivity for mild steatosis [3,4]. CAP, measured through vibration-controlled transient elastography (VCTE), enables quantitative assessment of hepatic fat content and can be combined with liver stiffness measurement during the same examination [59]. VCTE-based elastography allows for not only fibrosis assessment but also non-invasive steatosis recognition through CAP. MRI-PDFF represents the reference standard for steatosis quantification but is less accessible and more costly [60]. In lean patients, where steatosis may be mild or patchy, MRI-PDFF may offer advantages over ultrasound-based methods, although cost-effectiveness analyses are needed (Table 2).

### 4.4. Fibrosis Risk Stratification

Liver fibrosis is the strongest predictor of adverse outcomes in MASLD, including liver-related mortality, cardiovascular events, and overall survival [65]. Identification of patients with significant fibrosis (≥F2) or advanced fibrosis (≥F3) is therefore a clinical priority. Non-invasive fibrosis assessment has advanced, with validated serum biomarkers and imaging-based elastography techniques now widely used [3,4].

Serum-based scores, including the Fibrosis-4 (FIB-4) index and the NAFLD Fibrosis Score, incorporate routine laboratory and clinical variables to estimate fibrosis probability [62,63]. These tools perform reasonably well in general MASLD populations but have been less extensively validated in lean cohorts. FIB-4 may be affected by age and normal aminotransferases, and lean-specific validation remains limited. Enhanced fibrosis markers, such as the Enhanced Liver Fibrosis (ELF) test, may offer improved performance as second-line testing, although additional validation in lean populations is required [3,4].

Liver stiffness measurement through VCTE or magnetic resonance elastography (MRE) enables direct quantitative fibrosis assessment with high accuracy for advanced disease [61]. Liver stiffness is less affected by obesity-related technical failure in lean patients than in severe obesity, but it may be influenced by inflammation, cholestasis, congestion, and food intake. MRE has high diagnostic accuracy but is limited by cost and availability.

#### Histopathological Diagnosis

Histopathology remains the reference standard for confirming steatohepatitis and staging fibrosis, although liver biopsy is not required for most patients with suspected MASLD [3,4,66]. Under current terminology, MASH corresponds to the inflammatory and hepatocellular injury phenotype previously termed NASH. Histological assessment typically evaluates macrovesicular steatosis, lobular inflammation, hepatocellular ballooning, and fibrosis [66]. Disease activity is often described using the NAFLD Activity Score, which grades steatosis, lobular inflammation, and ballooning, whereas fibrosis is staged separately from F0 to F4 [66].

In lean MASLD, histology may be particularly useful when diagnosis is uncertain, aminotransferases are normal, but non-invasive tests suggest significant fibrosis, secondary causes remain possible, or pharmacotherapy/trial eligibility depends on MASH and fibrosis stage confirmation. However, biopsy has limitations, including invasiveness, sampling variability, interobserver variability, cost, and patient preference. Therefore, biopsy should be reserved for select cases in which histological confirmation is likely to change management [3,4].

### 4.5. Exclusion of Secondary Causes

In lean patients, the differential diagnosis of hepatic steatosis is broader and warrants careful exclusion of secondary causes. Beyond standard evaluation for alcohol use, viral hepatitis, and autoimmune liver disease, clinicians should consider causes that may be particularly relevant in normal-weight individuals, including HIV-associated lipodystrophy, lysosomal acid lipase deficiency (LAL-D), familial hypobetalipoproteinemia, abetalipoproteinemia, medication-induced hepatic steatosis, endocrine disorders, malnutrition, rapid weight loss, and environmental toxin exposures [3,4,19,67] (Table 3).

### 4.6. Proposed Diagnostic Approach

Given the challenges outlined above, a systematic approach to lean MASLD diagnosis is warranted. Clinicians should maintain a high index of suspicion in patients with unexplained transaminase elevation, fatty liver on imaging, metabolic risk factors, increased waist circumference, type 2 diabetes, prediabetes, dyslipidemia, hypertension, sarcopenia, or family history of advanced liver disease, regardless of BMI. Assessment should include measurement of waist circumference, evaluation for cardiometabolic comorbidities, exclusion of secondary causes, and stratification of fibrosis risk (Table 4).

## 5. Clinical Outcomes of Lean Versus Non-Lean MASLD

Lean MASLD generally carries greater risks than non-steatotic liver disease, but comparisons with overweight or obese MASLD are heterogeneous. Some cohorts report higher liver-related events and mortality in lean MASLD, whereas others show comparable hepatic outcomes but lower cardiovascular event incidence than non-lean MASLD [8,9,10,68,69,70]. These differences likely reflect variations in case definitions, fibrosis burden at diagnosis, genetic background, body composition, sarcopenia, competing risks, and ascertainment bias. Therefore, lean MASLD should not be interpreted as simply “milder fatty liver.” Instead, it represents a distinct phenotype with potentially substantial hepatic risk despite normal BMI (Table 5).

### 5.1. Liver-Related Outcomes

Compared with lean individuals without steatosis, patients with lean MASLD are at greater risk of liver-related outcomes. Compared with non-lean MASLD, however, the direction and magnitude of risk vary by cohort. This suggests that “lean” status modifies, but does not eliminate, the prognostic significance of hepatic steatosis, steatohepatitis, and fibrosis [8,9,10,69,70].

The most comprehensive prognostic data include multicohort and large database analyses. Huo et al. reported that lean MASLD was associated with a higher risk of liver-related events, all-cause mortality, liver-related mortality, and cardiovascular mortality compared with non-lean MASLD [8]. A large propensity-matched TriNetX cohort study similarly found that lean MASLD was associated with worse hepatic outcomes compared to non-lean, overweight, and obese MASLD groups, including fibrosis, cirrhosis, portal hypertensive complications, HCC, liver transplantation, and mortality [9]. Meta-analytic data also suggest higher all-cause and liver-related mortality in lean compared with non-lean NAFLD/MASLD, although heterogeneity and residual confounding are important limitations [68].

Other cohorts have reported more nuanced findings. A Japanese multicenter study found that lean MASLD had a higher liver-related risk than normal liver controls but comparable or variable risk compared with non-lean MASLD [70]. These findings indicate that lean MASLD should not be dismissed as clinically mild and that lean versus non-lean comparisons should be interpreted in light of baseline fibrosis, body composition, metabolic risk, and study design.

### 5.2. Cardiovascular Outcomes

MASLD is strongly associated with cardiovascular disease, a leading cause of death in this population [71]. The relationship between lean MASLD and cardiovascular risk is complex and conflicting across studies. Some population-based cohorts report lower cardiovascular event incidence in lean MASLD compared with non-lean MASLD, potentially reflecting lower rates of obesity, diabetes, hypertension, and dyslipidemia [8,70]. However, other clinical cohorts have reported higher rates of composite cardiovascular events, cerebrovascular events, heart failure, or cardiovascular-related mortality among lean MASLD patients [70,72].

A Veterans Affairs cohort study of patients with compensated MASLD cirrhosis found that lean MASLD cirrhosis was associated with higher all-cause mortality and cardiovascular-related mortality despite a lower prevalence of diabetes and a lower risk of hepatic decompensation compared with non-lean MASLD cirrhosis [72].

These discrepant findings likely reflect differences in study design, population characteristics, adjustment strategies, competing risks, and genetic modifiers, such as TM6SF2. Prospective studies with adjudicated cardiovascular endpoints are needed to clarify cardiovascular risk in lean MASLD.

### 5.3. Extrahepatic Manifestations

Beyond liver and cardiovascular outcomes, MASLD is associated with extrahepatic conditions including type 2 diabetes, chronic kidney disease, atrial fibrillation, heart failure, and extrahepatic malignancies [3,4,71]. Whether lean MASLD confers similar extrahepatic risk has not been systematically studied. Limited data suggest that lean MASLD patients may have lower absolute rates of diabetes and chronic kidney disease compared with their obese counterparts, but a higher risk compared with lean individuals without steatosis. Shared pathways—including insulin resistance, inflammation, visceral adiposity, sarcopenia, and dysbiosis—provide biological plausibility for extrahepatic risk even in the absence of obesity.

### 5.4. Mortality

All-cause mortality is increased in MASLD compared with that in the general population, driven primarily by cardiovascular, cancer-related, and liver-related deaths [1,71]. The impact of lean status on survival has been clarified by recent studies showing increased mortality in lean MASLD compared with non-lean MASLD in some cohorts [8,9,69,72]. Mechanisms underlying this paradoxically higher mortality rate may include reverse causation, sarcopenia, delayed diagnosis, genetic predisposition, lower physiological reserve, and unmeasured confounding by body composition variables not captured by BMI.

Further research with standardized definitions, adequate follow-up, and rigorous adjustment for confounders—including body composition measures beyond BMI—is needed to clarify the prognostic implications of lean MASLD; key cohort studies and meta-analyses evaluating these outcomes are summarized in Table 6.

## 6. Management of Lean MASLD

### 6.1. Lifestyle Modifications

Lifestyle interventions, including dietary modification, increased physical activity, and body composition optimization, are the cornerstones of MASLD therapy across the BMI spectrum [3,4]. In lean patients, the therapeutic target is not necessarily weight loss but rather improvements in metabolic health, visceral adiposity, muscle mass, insulin sensitivity, and hepatic fat content. The AGA Clinical Practice Update on lean NAFLD emphasizes that modest weight reduction may reduce hepatic steatosis in select lean patients, but management should be individualized and avoid excessive weight loss in patients with low BMI or sarcopenia [19].

Dietary recommendations emphasize reducing refined carbohydrates and added sugars, particularly fructose-containing beverages, limiting saturated fat intake, and adopting Mediterranean-style eating patterns rich in monounsaturated fatty acids, fiber, and plant-based foods [48,49]. Exercise, including both aerobic and resistance training, has independent benefits for MASLD irrespective of weight change. Resistance training is particularly relevant in lean patients with sarcopenia or low muscle reserve [51,68].

#### Adjunctive Non-Pharmacological Approaches

Several adjunctive non-pharmacological strategies have been investigated in MASLD, although evidence specific to lean MASLD remains limited. Coffee consumption has been associated with a lower risk of advanced fibrosis and liver-related outcomes in observational studies and may be reasonable when not contraindicated [74]. Omega-3 fatty acids may improve hypertriglyceridemia and liver fat but have not consistently demonstrated histological benefit [3,4]. Whey protein supplementation may support muscle protein synthesis and improve body composition in patients with low protein intake or sarcopenia, particularly when combined with resistance exercise [54,75]. Vitamin D repletion should be considered in deficient patients for general musculoskeletal health, although evidence for direct MASLD improvement is inconsistent [53]. Probiotics, synbiotics, and select phytotherapeutic compounds have been studied, but results are heterogeneous, and product quality, dosing, drug interactions, and hepatotoxicity risk remain important concerns [47,76]. These approaches should be viewed as adjunctive rather than disease-modifying, and they should not replace fibrosis risk stratification, cardiometabolic optimization, or evidence-based pharmacotherapy when indicated.

### 6.2. Cardiometabolic Risk Optimization

Given the association between MASLD and cardiovascular disease, comprehensive management of cardiometabolic risk factors is essential. This includes glycemic control optimization in patients with diabetes or prediabetes, hypertension treatment, and dyslipidemia management with statins or other lipid-lowering agents as indicated [3,4,77].

Statins are safe in patients with MASLD and should not be withheld solely because of mild aminotransferase elevation [3,4,78]. Observational studies and meta-analyses suggest that statins reduce cardiovascular risk in MASLD and may be associated with a lower risk of hepatic decompensation, HCC, or fibrosis progression, although these hepatic benefits require cautious interpretation, as much of the evidence is non-randomized [79]. In lean MASLD patients with dyslipidemia, statin therapy should be initiated according to established cardiovascular risk algorithms.

The 2025 American Diabetes Association (ADA) Standards of Care emphasize that comprehensive cardiometabolic risk management is warranted in patients with MASLD, with attention to glycemic control, lipid management, blood pressure optimization, and obesity- or body-composition-related risk when applicable [77].

### 6.3. Pharmacotherapy for MASH

The therapeutic landscape for MASH has been transformed by the accelerated approval of two agents for adults with noncirrhotic MASH/NASH with moderate to advanced fibrosis, to be used with diet and exercise [14,15,16,17].

Resmetirom, an oral, liver-directed thyroid hormone receptor-β-selective agonist, received accelerated FDA approval in March 2024 for adults with noncirrhotic NASH/MASH with moderate to advanced fibrosis [14,15]. In the phase 3 MAESTRO-NASH trial, 52 weeks of resmetirom were superior to placebo for both MASH/NASH resolution without worsening of fibrosis and fibrosis improvement by at least one stage without worsening of steatohepatitis [14]. Resmetirom also improved atherogenic lipid parameters, including LDL cholesterol, triglycerides, and lipoprotein(a), with generally neutral effects on body weight [14].

Treatment initiation does not necessarily require liver biopsy when clinical and non-invasive tests support a noncirrhotic MASH diagnosis with moderate to advanced fibrosis, although local practice and payer requirements may vary [3,4,15]. Resmetirom is not indicated for decompensated cirrhosis, and careful assessment for other active liver diseases is important. Common adverse events include gastrointestinal symptoms, particularly diarrhea and nausea [14,15].

Semaglutide, a glucagon-like peptide-1 receptor agonist, received accelerated FDA approval in August 2025 for adults with noncirrhotic MASH with moderate to advanced fibrosis, to be used with diet and exercise [16,17]. In the phase 3 ESSENCE trial, 2.4 mg of semaglutide once weekly improved histological outcomes in patients with MASH and moderate to advanced fibrosis, including MASH resolution without worsening of fibrosis and fibrosis improvement without worsening of MASH [16]. Semaglutide also produced substantial weight loss and improved metabolic parameters [16].

Semaglutide is not approved for MASH cirrhosis. Gastrointestinal symptoms are common and usually dose-related. Clinicians should monitor for dehydration-related kidney injury, gallbladder disease, pancreatitis risk, and excessive weight or lean mass loss, particularly in patients who are already lean or sarcopenic [16,17] (Table 7).

### 6.4. Applicability of Approved Pharmacotherapies to Lean MASLD

The applicability of resmetirom and semaglutide to lean MASLD populations remains uncertain because pivotal trials enrolled predominantly overweight or obese populations [14,16]. Lean patients were not necessarily excluded but were underrepresented, and dedicated lean MASLD subgroup analyses are limited.

Several considerations are relevant to lean MASLD pharmacotherapy. Resmetirom may be particularly suitable for select lean patients because its efficacy is not primarily dependent on weight loss and it is generally weight-neutral, which may be important in patients with normal BMI, low muscle mass, or sarcopenia [14]. Its lipid-lowering effects may also be advantageous in lean patients with atherogenic dyslipidemia.

Semaglutide may be beneficial in lean patients with insulin resistance, prediabetes, type 2 diabetes, or broader cardiometabolic risk. However, its weight-reducing effect may be undesirable in patients with low BMI, sarcopenia, frailty, or inadequate protein intake [16]. In such patients, treatment should be individualized, with nutritional status monitoring, resistance exercise, protein intake, and objective or functional muscle mass measures when feasible [54,68].

At present, there is insufficient evidence to recommend one approved agent over another specifically for lean MASLD. Therapy should be individualized according to fibrosis stage, metabolic phenotype, comorbidities, body composition, patient preference, tolerability, and access. Dedicated prospective studies and prespecified subgroup analyses in lean MASLD are urgently needed.

### 6.5. Emerging Therapies

Several investigational agents may further expand the therapeutic landscape for MASH. Tirzepatide, a dual glucose-dependent insulinotropic polypeptide/glucagon-like peptide-1 receptor agonist, demonstrated significant histological improvement in a phase 2 MASH trial, including higher rates of MASH resolution and fibrosis improvement compared with placebo [80]. Other agents under investigation include dual GLP-1/glucagon receptor agonists, FGF21 analogs, PPAR agonists, and combination strategies targeting complementary metabolic, inflammatory, and fibrotic pathways [81]. However, lean MASLD-specific data remain limited, and future trials should include prespecified analyses by BMI, visceral adiposity, sarcopenia, diabetes status, and genetic risk.

### 6.6. Addressing Sarcopenia

Recognition and management of sarcopenia are particularly relevant in lean MASLD, given its association with disease severity and adverse outcomes. Assessment of muscle mass and function using bioelectrical impedance analysis, dual-energy X-ray absorptiometry, computed tomography, grip strength, or gait speed should be considered in at-risk patients, following consensus criteria when feasible [68].

Interventions to preserve or restore muscle mass include resistance training, adequate protein intake, and vitamin D optimization when deficient [53,54,68]. Multidisciplinary collaboration with dietitians and exercise professionals may optimize outcomes. In patients receiving semaglutide, monitoring for excessive weight loss and lean mass loss is particularly important [16].

### 6.7. Treatment Response Monitoring

For patients receiving pharmacotherapy, treatment response should be assessed using non-invasive tests and clinical parameters. Liver biochemistry, metabolic risk factors, weight trajectory, nutritional status, and tolerability should be monitored longitudinally. For resmetirom and semaglutide, improvements in aminotransferases, liver stiffness measurement, MRI-PDFF, ELF, or other validated non-invasive markers may support therapeutic response, although histological response cannot be assumed from biochemical improvement alone [3,4,14,16].

In lean patients, monitoring should also include assessment for unintended weight loss, sarcopenia progression, inadequate protein intake, or functional decline. This is especially important when using weight-reducing therapies.

### 6.8. Surveillance for Complications

Patients with advanced fibrosis or cirrhosis require surveillance and management according to standard chronic liver disease guidelines. HCC surveillance is recommended in patients with cirrhosis, typically every six months with abdominal ultrasound with or without alpha-fetoprotein measurement [81]. The role of routine HCC surveillance in noncirrhotic advanced fibrosis is debated and should be individualized based on guideline recommendations and patient-level risk [81].

Screening for esophageal varices should be performed in patients with cirrhosis or clinically significant portal hypertension, with the use of non-invasive criteria to identify patients who may safely avoid endoscopy when appropriate [3,4]. Lean MASLD patients with significant fibrosis or cirrhosis should be managed similarly to non-lean MASLD patients with equivalent fibrosis stage, while also addressing sarcopenia and body composition risk (Table 8).

## 7. Future Directions

Several priorities for future research and clinical practice can be identified based on current evidence.

First, large prospective cohort studies with standardized definitions of lean MASLD are needed to clarify incidence, prevalence, and temporal trends across diverse populations. Harmonization of BMI thresholds, metabolic criteria, alcohol thresholds, and steatosis detection methods will facilitate cross-study comparisons.

Second, a deeper understanding of the biological heterogeneity underlying lean MASLD—including genetic, metabolic, microbiome, dietary, sleep-related, and environmental contributors—will inform risk stratification and therapeutic targeting. Multi-omics approaches integrating genomics, metabolomics, transcriptomics, and microbiome profiling hold promise.

Third, diagnostic tools optimized for lean populations are needed. Existing non-invasive tests were largely developed and validated in broader MASLD or NAFLD cohorts, and lean-specific thresholds for CAP, liver stiffness, ELF, and composite scores require further study.

Fourth, lean patients should be intentionally included in clinical trials of MASH pharmacotherapies. Both resmetirom and semaglutide have changed MASH management, but efficacy and safety data in lean patients remain limited [14,16]. Dedicated studies or prespecified subgroup analyses should evaluate treatment response by BMI, waist circumference, visceral adiposity, sarcopenia, diabetes status, and genetic risk.

Finally, public health initiatives should emphasize that normal BMI does not exclude clinically important steatotic liver disease. Educational programs and clinical decision support tools should encourage fibrosis risk assessment in lean individuals with hepatic steatosis, metabolic risk factors, or unexplained liver test abnormalities.

## 8. Conclusions

Lean MASLD is an underrecognized but clinically important form of steatotic liver disease. Even with normal BMI, affected individuals may have significant metabolic dysfunction, visceral adiposity, sarcopenia, advanced fibrosis, and increased liver-related and overall mortality. Diagnosis is often delayed because BMI underestimates risk and normal aminotransferases do not exclude MASH or clinically significant fibrosis. A systematic approach incorporating steatosis confirmation, exclusion of secondary causes, fibrosis risk stratification, and body composition assessment is needed. Although resmetirom and semaglutide have expanded treatment options for noncirrhotic MASH with moderate to advanced fibrosis, their role in lean MASLD requires further study. Greater awareness, earlier risk stratification, individualized management, and lean-specific research are needed to improve outcomes.

## Figures and Tables

**Table 1:** Key pathophysiological mechanisms in lean MASLD.

Mechanism	Contribution to Lean MASLD	Clinical Implications
Visceral adiposity	Normal BMI may coexist with increased visceral adipose tissue, promoting insulin resistance, inflammation, and hepatic lipid accumulation [25,32,33,34].	Waist circumference and body composition may be more informative than BMI alone.
Insulin resistance	Increases adipose lipolysis, free fatty acid flux to the liver, and de novo lipogenesis [30,31,38].	Lean patients should still be screened for prediabetes, diabetes, dyslipidemia, and hypertension.
Genetic susceptibility	Variants such as PNPLA3, TM6SF2, MBOAT7, GCKR, and HSD17B13 may predispose individuals to hepatic steatosis, MASH, fibrosis, or HCC independent of obesity [40,41,42,43,44,45].	Routine genetic testing is not currently recommended, but genetic enrichment may explain severe disease in select lean patients [3,4,19].
Sarcopenia	Reduced skeletal muscle mass impairs glucose disposal and worsens metabolic flexibility, contributing to insulin resistance and fibrosis risk [35,36,37].	Resistance training, protein optimization, and sarcopenia screening are important in select patients.
Gut microbiome dysbiosis	Altered microbial composition may increase intestinal permeability, endotoxemia, bile acid signaling disruption, and hepatic inflammation [46,47].	Microbiome-directed therapies remain investigational.
Dietary and lifestyle exposures	High fructose intake, refined carbohydrates, saturated fat, sedentary behavior, poor sleep, and circadian disruption may promote hepatic steatosis even without obesity [38,39,50,52].	Lifestyle counseling should focus on metabolic quality and body composition, not only weight loss.
Vitamin D deficiency and endocrine factors	Vitamin D deficiency, hypothyroidism, and polycystic ovary syndrome may contribute to metabolic dysfunction and steatosis [3,4,53].	Select patients should undergo targeted evaluation based on clinical context.

**Table 2:** Non-invasive tests for steatosis and fibrosis assessment in lean MASLD.

Test	Primary Use	Strengths	Limitations in Lean MASLD
Ultrasound	Hepatic steatosis detection	Widely available, inexpensive, and no radiation exposure [3,4].	Reduced sensitivity for mild steatosis; operator-dependent; cannot reliably stage fibrosis.
CAP by VCTE	Steatosis quantification	Point-of-care assessment; can be combined with liver stiffness measurement [59,61].	Cutoffs vary by population; mild steatosis may be missed; lean-specific thresholds remain insufficiently validated.
MRI-PDFF	Quantitative liver fat measurement	Most accurate non-invasive method for steatosis quantification; useful in mild or equivocal steatosis [60].	Higher cost and limited availability.
FIB-4	First-line fibrosis triage	Simple, inexpensive, and uses age, AST, ALT, and platelet count [3,62].	May be affected by age and normal aminotransferases; lean-specific validation remains limited.
NAFLD Fibrosis Score	Fibrosis risk estimation	Historically validated in NAFLD cohorts [63].	Includes BMI and diabetes variables; performance may differ in lean populations.
ELF test	Serologic fibrosis assessment	Reflects extracellular matrix turnover; useful as second-line testing [3,4].	Cost and availability vary; lean-specific cutoffs require further validation.
VCTE liver stiffness measurement	Fibrosis assessment	Non-invasive, rapid, widely used; less affected by BMI in lean patients than in severe obesity [61].	Affected by inflammation, congestion, cholestasis, and food intake; optimal lean-specific thresholds remain uncertain.
MRE	Advanced fibrosis assessment	High diagnostic accuracy for fibrosis; useful when VCTE is indeterminate [61,64].	Costly and less available.

**Table 3:** Secondary causes and alternative diagnoses to consider in lean patients with hepatic steatosis.

Category	Examples	Suggested Evaluation
Alcohol-related liver disease	Alcohol intake above accepted MASLD thresholds or binge pattern drinking	Detailed alcohol history; validated alcohol use screening tools; biomarkers when clinically indicated [2,3,4]
Viral hepatitis	Hepatitis B, hepatitis C	HBsAg, anti-HBc, anti-HBs, anti-HCV with reflex HCV RNA when positive [3,4]
Autoimmune liver disease	Autoimmune hepatitis, primary biliary cholangitis, primary sclerosing cholangitis	ANA, ASMA, IgG, AMA, alkaline phosphatase pattern, and MRCP when cholestatic disease is suspected [3,4]
Medication-induced steatosis/steatohepatitis	Amiodarone, methotrexate, tamoxifen, corticosteroids, valproate, some antiretroviral agents, irinotecan	Medication history, duration, dose, temporal relationship, and consideration of alternatives [3,4,19]
Endocrine/metabolic disorders	Hypothyroidism, polycystic ovary syndrome, hypopituitarism, uncontrolled diabetes	TSH, HbA1c, fasting glucose, and clinical endocrine evaluation when indicated [3,4]
Genetic lipid disorders	Familial hypobetalipoproteinemia, abetalipoproteinemia	Fasting lipid profile, apolipoprotein B, family history, and genetic testing in select cases [19]
Lysosomal acid lipase deficiency	Unexplained steatosis with dyslipidemia, hepatomegaly, elevated aminotransferases, low HDL cholesterol, high LDL cholesterol, or premature atherosclerosis	Dried blood spot lysosomal acid lipase enzyme activity; confirmatory LIPA genetic testing when enzyme activity is low [67]
Lipodystrophy	Generalized or partial loss of subcutaneous fat with severe insulin resistance, hypertriglyceridemia, or diabetes	Clinical examination, metabolic profile, leptin level in select cases, and specialist referral [19]
Malnutrition or rapid weight loss	Starvation, eating disorders, gastrointestinal surgery, parenteral nutrition	Nutritional history, weight trajectory, albumin/prealbumin when appropriate, and dietitian assessment [3,4]
Wilson disease in select patients	Young age, unexplained liver disease, neuropsychiatric features	Ceruloplasmin, 24 h urinary copper, slit-lamp examination, and ATP7B testing when indicated [3,4]

**Table 4:** Proposed stepwise approach to diagnosis and risk stratification in lean MASLD.

Step	Evaluation	Purpose
1	History, physical examination, BMI, waist circumference, medication review, alcohol history, family history	Identify metabolic risk factors, central adiposity, secondary causes, and inherited/metabolic clues
2	Liver biochemistry, platelet count, fasting glucose or HbA1c, lipid profile, renal function, and thyroid-stimulating hormone when clinically indicated	Assess liver injury, cardiometabolic dysfunction, and conditions that may contribute to steatosis; normal aminotransferases do not exclude MASH or fibrosis [3,19,55]
3	Steatosis assessment using ultrasound, CAP by VCTE, or MRI-PDFF	Confirm hepatic steatosis; MRI-PDFF may be useful when ultrasound is equivocal or steatosis is mild [59,60,61]
4	Exclusion of secondary causes: alcohol, medications, viral hepatitis, autoimmune disease, endocrine disease, lipodystrophy, LAL-D, hypobetalipoproteinemia, Wilson disease in select younger patients	Rule out alternative or coexisting etiologies, particularly in lean individuals [3,4,19,67]
5	First-tier fibrosis assessment: FIB-4	Initial fibrosis triage; low < 1.3, indeterminate 1.3–2.67, high > 2.67, with age-adjusted interpretation in older adults [3,62]
6	Second-tier fibrosis assessment if FIB-4 is indeterminate/high or clinical suspicion persists despite low FIB-4: VCTE liver stiffness measurement, ELF test, or MRE	Refine fibrosis risk; persistent concern should prompt elastography even with normal aminotransferases [3,4,61]
7	Sarcopenia/body composition assessment in select patients: grip strength, gait speed, DXA, bioelectrical impedance, or CT-based muscle area when available	Identify high-risk lean patients with low muscle reserve or sarcopenic phenotype [35,36,37,68]
8	Liver biopsy in select cases	Confirm MASH, stage fibrosis, resolve diagnostic uncertainty, or determine trial eligibility [3,4,66]

**Table 5:** Comparison of lean MASLD and non-lean MASLD.

Feature	Lean MASLD	Non-Lean MASLD
BMI	Normal BMI, typically <25 kg/m^2^ in Western populations and <23 kg/m^2^ in Asian populations [18,19].	Overweight or obese BMI range.
Body composition	May have increased visceral adiposity, low muscle mass, or sarcopenia despite normal BMI [25,32,33,34,35,36,37].	Generalized and central adiposity are more common.
Metabolic profile	May have subtle insulin resistance, dyslipidemia, hypertension, or prediabetes; overt metabolic syndrome may be absent [6,7,11].	Higher prevalence of type 2 diabetes, metabolic syndrome, hypertension, and dyslipidemia.
Genetic contribution	Potentially stronger relative contribution of PNPLA3, TM6SF2, GCKR, MBOAT7, and other variants [40,41,42,43,44,45].	Genetic risk interacts with obesity and metabolic syndrome.
Diagnosis	Often delayed because clinicians may not suspect MASLD in normal-weight individuals. Normal aminotransferases are possible [3,11,19,55].	More likely to be screened because obesity and diabetes prompt clinical suspicion.
Fibrosis risk	Non-negligible; may be advanced at diagnosis in some cohorts [9,23,26,56,68].	Strongly related to diabetes, obesity severity, and metabolic syndrome.
Cardiovascular risk	Conflicting evidence; some population cohorts report lower CVD incidence, whereas other clinical cohorts report higher CVD events or cardiovascular mortality [8,69,70].	CVD is a leading cause of morbidity and mortality [71].
Management emphasis	Body composition, sarcopenia, visceral adiposity, cardiometabolic risk, and careful pharmacotherapy selection.	Weight loss, cardiometabolic control, and fibrosis-directed therapy.
Pharmacotherapy considerations	Weight-neutral therapy may be attractive in patients with low BMI or sarcopenia; GLP-1 receptor agonist therapy requires monitoring for excessive weight or lean mass loss [14,16].	Weight-lowering therapies may provide broader metabolic benefits.

**Table 6:** Major cohort studies and meta-analyses evaluating outcomes in lean MASLD/lean NAFLD.

Study	Year	Population/Sample	Lean Definition	Key Findings	Limitations
Ye et al. [5]	2020	Systematic review/meta-analysis of non-obese or lean NAFLD	BMI-based definitions varying by region	Lean/non-obese NAFLD represented a substantial proportion of NAFLD globally and was associated with metabolic and hepatic risk.	Heterogeneous definitions and diagnostic methods; many studies used NAFLD rather than MASLD criteria.
Ha et al. [69]	2023	Meta-analysis of cohort studies comparing lean versus non-lean NAFLD	BMI-based lean definitions	Lean NAFLD was associated with higher all-cause and liver-related mortality compared with non-lean NAFLD.	Residual confounding; variable adjustment for fibrosis and metabolic risk; NAFLD-era terminology.
Wakabayashi et al. [70]	2024	Large Japanese multicenter cohort	BMI-based lean MASLD definition	Lean MASLD had higher liver-related risk than normal liver controls and variable risk compared with non-lean MASLD.	Population-specific findings; generalizability outside of Japan may be limited.
Njei et al. [11]	2025	NHANES 2017–2020 U.S. adults	Normal BMI with MASLD criteria	Lean MASLD was prevalent among U.S. adults, and a substantial proportion of cases were undiagnosed.	Cross-sectional design; limited longitudinal outcomes.
Huo et al. [8]	2026	UK Biobank, Kailuan, and China Kadoorie Biobank multicohort analysis	BMI-based lean MASLD definition	Lean MASLD was associated with higher liver-related events, liver-related mortality, all-cause mortality, and CVD mortality compared with non-lean MASLD.	Potential residual confounding; population heterogeneity; competing risks.
Chowdhary et al. [9]	2026	Large propensity-matched TriNetX MASLD cohort	BMI-based lean MASLD definition	Lean MASLD was associated with higher odds of fibrosis, cirrhosis, portal hypertensive complications, HCC, liver transplantation, and mortality.	EHR coding-based definitions; potential misclassification; limited histological confirmation.
Al Ta’ani et al. [71]	2026	Propensity-matched multicenter cohort	BMI-based lean MASLD definition	Lean MASLD was associated with higher rates of cardiovascular and cerebrovascular events and all-cause mortality.	Observational design; endpoint definitions may vary; residual confounding possible.
Njei et al. [73]	2025	Veterans Affairs cohort of compensated MASLD cirrhosis	BMI-based lean definition	Lean MASLD cirrhosis was associated with higher all-cause and cardiovascular-related mortality despite lower hepatic decompensation risk.	Predominantly male VA population; cirrhosis cohort; observational design.

**Table 7:** Comparison of currently approved pharmacotherapies for noncirrhotic MASH with moderate to advanced fibrosis.

Feature	Resmetirom	Semaglutide
Drug class	Liver-directed thyroid hormone receptor-β agonist	Glucagon-like peptide-1 receptor agonist
Route	Oral, once daily	Subcutaneous injection, once weekly
Regulatory indication	Adults with noncirrhotic NASH/MASH with moderate to advanced fibrosis, used with diet and exercise [15]	Adults with noncirrhotic MASH with moderate to advanced fibrosis, used with diet and exercise [17]
Main trial evidence	MAESTRO-NASH [14]	ESSENCE [16]
Histological effect	Improves MASH/NASH resolution and fibrosis improvement compared with placebo [14]	Improves MASH resolution and fibrosis improvement compared with placebo [16]
Weight effect	Generally weight-neutral [14]	Produces substantial weight loss [16]
Metabolic effects	Improves LDL cholesterol, triglycerides, and lipoprotein(a) [14]	Improves glycemic control, insulin resistance, and weight-related cardiometabolic risk [16]
Potential role in lean MASLD	Potentially attractive when weight loss is undesirable or sarcopenia is present	May benefit patients with diabetes, insulin resistance, or broader cardiometabolic risk, but requires monitoring for excessive weight loss and lean mass loss
Cautions	Avoid use in decompensated cirrhosis; assess for other active liver diseases and monitor tolerability [14,15]	Not approved for MASH cirrhosis; need to monitor gastrointestinal tolerance, renal function during dehydration, gallbladder disease, pancreatitis risk, and lean mass [16,17]
Evidence gap	Lean-MASLD-specific efficacy and safety data are limited	Lean-MASLD-specific efficacy and safety data are limited

**Table 8:** Practical management algorithm for lean MASLD patients.

Clinical Step	Recommended Actions	Rationale
1. Confirm steatosis and MASLD criteria	Document hepatic steatosis by ultrasound, CAP/VCTE, MRI-PDFF, or histology; confirm at least one cardiometabolic risk factor.	Ensures alignment with current MASLD nomenclature [2,3,4].
2. Exclude secondary causes	Assess alcohol use, medications, viral hepatitis, autoimmune liver disease, endocrine/metabolic disorders, LAL-D, lipid disorders, and lipodystrophy when clinically indicated.	Lean patients have a broader differential diagnosis [3,4,19,67].
3. Perform first-tier fibrosis triage	Calculate FIB-4 using age, AST, ALT, and platelet count.	Low-cost initial risk stratification [3,62].
4. Perform second-tier testing when indicated	Use VCTE liver stiffness measurement, ELF, or MRE if FIB-4 is indeterminate/high or suspicion persists despite normal aminotransferases.	Normal enzymes do not exclude MASH or fibrosis [3,4,55].
5. Assess body composition and sarcopenia risk	Consider waist circumference, grip strength, gait speed, DXA, BIA, or CT-based muscle assessment in select patients.	Sarcopenia may worsen insulin resistance and fibrosis risk [35,36,37,68].
6. Treat low fibrosis risk disease	Lifestyle optimization, Mediterranean-style diet, reduced refined carbohydrates, aerobic/resistance exercise, and cardiometabolic risk control.	Improves steatosis and metabolic health without excessive weight loss [48,49,51].
7. Treat suspected F2–F3 disease	Refer to hepatology; consider pharmacotherapy individualized to phenotype. Resmetirom may be favored when weight loss is undesirable; semaglutide may be favored when diabetes or insulin resistance predominates.	Approved therapies exist, but lean-specific evidence is limited [14,15,16,17].
8. Manage cirrhosis if present	HCC surveillance, portal hypertension assessment, variceal screening when indicated, vaccination, nutrition optimization, and transplant referral when appropriate.	Fibrosis stage remains the strongest determinant of liver-related outcomes [3,4,65,81].

## Data Availability

No new data were created or analyzed in this study. Data sharing is not applicable to this article.

## Abbreviations

The following abbreviations are used in this manuscript:

ADA	American Diabetes Association
ALT	Alanine aminotransferase
AMA	Antimitochondrial antibody
ANA	Antinuclear antibody
ASMA	Anti-smooth muscle antibody
AST	Aspartate aminotransferase
BIA	Bioelectrical impedance analysis
BMI	Body mass index
CAP	Controlled attenuation parameter
ChREBP	Carbohydrate-responsive element-binding protein
CVD	Cardiovascular disease
DNL	De novo lipogenesis
DXA	Dual-energy X-ray absorptiometry
ELF	Enhanced Liver Fibrosis test
FDA	U.S. Food and Drug Administration
FIB-4	Fibrosis-4 index
GCKR	Glucokinase regulator
GIP	Glucose-dependent insulinotropic polypeptide
GLP-1	Glucagon-like peptide-1
HCC	Hepatocellular carcinoma
HDL	High-density lipoprotein
IgG	Immunoglobulin G
LAL-D	Lysosomal acid lipase deficiency
LDL	Low-density lipoprotein
LPS	Lipopolysaccharide
LSM	Liver stiffness measurement
MASH	Metabolic dysfunction-associated steatohepatitis
MASLD	Metabolic dysfunction-associated steatotic liver disease
MBOAT7	Membrane-bound O-acyltransferase domain-containing 7
MRCP	Magnetic resonance cholangiopancreatography
MRE	Magnetic resonance elastography
MRI-PDFF	Magnetic resonance imaging-derived proton density fat fraction
NAFLD	Nonalcoholic fatty liver disease
NASH	Nonalcoholic steatohepatitis
PNPLA3	Patatin-like phospholipase domain-containing 3
PPAR	Peroxisome proliferator-activated receptor
SREBP-1c	Sterol regulatory element-binding protein-1c
THR-β	Thyroid hormone receptor beta
TM6SF2	Transmembrane 6 superfamily member 2
TSH	Thyroid-stimulating hormone
VAT	Visceral adipose tissue
VCTE	Vibration-controlled transient elastography
